# AMPA Receptors in NG2 Glia Differently Affect Signal Transduction in the Hippocampus and Cerebellum

**DOI:** 10.1002/glia.70107

**Published:** 2025-12-02

**Authors:** Dario Tascio, Nehal Gebril, Ronald Jabs, Christian Henneberger, Christian Steinhäuser, Gerald Seifert

**Affiliations:** ^1^ Institute of Cellular Neurosciences I, Medical Faculty University of Bonn Bonn Germany; ^2^ German Center for Neurodegenerative Diseases (DZNE) Bonn Germany

**Keywords:** AMPA receptor, cerebellum, hippocampus, NG2 glia, patch clamp, subunit composition

## Abstract

Gray matter NG2 glia constitute a heterogeneous population of cells whose functions remain incompletely understood. In the hippocampus, Schaffer collaterals activate AMPA receptors (AMPARs) in NG2 glia, giving rise to small excitatory post‐synaptic currents (EPSCs). Climbing fibers of the cerebellum also form synapses with NG2 glia, although producing much larger EPSCs. We aimed to identify mechanisms generating these regional differences in the efficacy of neuron–glia synapses. Combined patch‐clamp and RT‐PCR analyses allowed for determining structural and functional differences of AMPARs expressed by the glial cells. Comparing pharmacological and molecular data in both regions revealed stronger expression of Ca^2+^ permeable AMPARs in cerebellar NG2 glia. Different expression patterns were found both for AMPAR subunits and their auxiliary proteins. Moreover, experiments using the low‐affinity AMPAR antagonist γ‐DGG pointed towards higher synaptic glutamate concentrations at cerebellar synapses, likely due to multivesicular release, which contributed to enhanced synaptic efficacy. Finally, we examined short‐term plasticity and showed that pre‐ and postsynaptic mechanisms contributed to paired‐pulse depression at climbing fiber–NG2 glia synapses. Together, our data provide new insights into the molecular and functional specialization of NG2 glia and improve our understanding of the mechanisms underlying neuron–glia synaptic signaling, by highlighting how region‐specific differences in AMPAR composition and presynaptic release properties shape this communication in the central nervous system.

## Graphical abstract

- Stronger expression of Ca^2+^ permeable AMPA receptors in cerebellar versus hippocampal NG2 glia.
- Different expression patterns of glial AMPA receptors and auxiliary subunits in both regions.
- Higher efficacy of neuron‐NG2 glia synapses in the cerebellum.

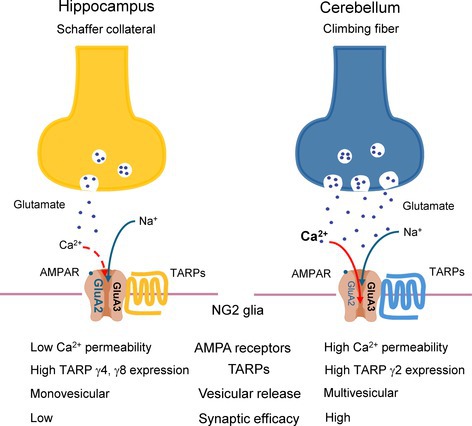

## 1 Introduction

AMPA‐type glutamate receptors (AMPARs) are ligand‐gated ion channels that mediate most of the fast excitatory synaptic transmission in the central nervous system (CNS). These receptors are homo‐ or heterotetrameric complexes composed of subunits GluA1‐4 which affect receptor kinetics, ion selectivity and their interactions with auxiliary proteins (Shepherd and Huganir 2007; Traynelis et al. 2010). The functional properties of AMPARs are largely determined by the presence or absence of the GluA2 subunit. Indeed, this subunit undergoes RNA editing, where a positively charged arginine residue (R) replaces a neutral glutamine (Q) at the Q/R site. Thus, when GluA2 is incorporated into the receptor, it renders it impermeable to Ca^2+^ ions and resistant to block by intracellular polyamines, resulting in characteristic outwardly rectifying current–voltage (I‐V) relationships (Bowie and Mayer 1995; Geiger et al. 1995; Swanson et al. 1997). In contrast, AMPARs lacking the GluA2 subunit are Ca^2+^‐permeable (CP‐AMPARs) and have a linear or inwardly rectifying I‐V curve. The diversity of AMPARs is further enhanced by auxiliary subunits, such as transmembrane AMPAR regulatory proteins (TARPs), which influence many aspects of receptor physiology, from trafficking to gating and Ca^2+^ permeability (Tomita et al. 2005; Jackson and Nicoll 2011). The composition of AMPARs varies across brain regions and cell types, and most cells express varying proportions of GluA2‐lacking and GluA2‐containing AMPARs, contributing to the functional specialization of synaptic signaling in different neuronal circuits (Greger et al. 2017).

In addition to their well‐established roles in neurons, AMPARs also mediate neuron–glia communication. Indeed, NG2 glia—also known as oligodendrocyte precursor cells (OPCs)—are unique in their ability to receive direct synaptic input from neurons (Bergles et al. 2000; Jabs et al. 2005). They constitute a heterogeneous cell population distributed throughout both white and gray matter of the CNS. In white matter, these cells serve as precursors for myelinating oligodendrocytes, contributing to the formation and maintenance of myelin sheaths during lifespan. In gray matter, most NG2 glia retain their precursor phenotype and engage in direct synaptic communication with neurons (Nishiyama et al. 2009). The synaptic properties of NG2 glia at glutamatergic synapses vary across different brain regions, reflecting the diversity of AMPAR composition and function. For example, in the hippocampus, NG2 glia are relatively homogenously distributed and express AMPARs with intermediate Ca^2+^ permeability involved in synaptic signaling, and undergo developmental regulation (Seifert et al. 2003; Hardt et al. 2021). In contrast, in the cerebellar molecular layer, NG2 glia are sparser, but individual NG2 glial cells receive dense innervation from multiple climbing fibers (CFs, Lin et al. 2005). CF inputs evoke strong AMPA‐mediated postsynaptic currents (PSCs) in NG2 glia, with amplitudes up to 10‐fold larger than those in the hippocampus. However, it is not yet clear whether the stronger synaptic input in cerebellar NG2 glia is solely due to the strong CF innervation or whether differences in AMPAR properties and additional synaptic mechanisms also play a role.

This study investigates mechanisms underlying the differences in synaptic efficacy of neuron‐NG2 glia synapses between the hippocampus and cerebellum. We examined whether the high Ca^2+^ permeability of AMPARs expressed by cerebellar NG2 glia is the primary determinant of the stronger synaptic transmission, and whether differences in AMPAR composition, along with multivesicular release, also contribute to the high synaptic efficacy in that region. Our results demonstrate that cerebellar NG2 glia synapses exhibit a higher proportion of CP‐AMPARs and larger glutamate transients in the synaptic cleft, which together produce a higher synaptic efficacy than in the hippocampus. AMPARs of cerebellar NG2 glia were Ca^2+^ permeable because CF‐evoked excitatory postsynaptic currents (CF‐EPSCs) showed inward rectification in the presence of intracellular spermine (Coombs et al. 2023). Furthermore, we analyzed the expression pattern of AMPAR subunits and their auxiliary proteins that contribute to the functional specialization of NG2 glia in the hippocampus and cerebellum. Finally, we examined short‐term plasticity at CF‐NG2 glia synapses, revealing both pre‐ and postsynaptic mechanisms that regulate synaptic dynamics. Our findings provide new insights into how NG2 glia integrate synaptic input in a region‐specific manner.

## 2 Methods

### 2.1 Animals and Ethical Approval

Male and female NG2‐EYFPki mice (Karram et al. 2008) of postnatal days (*p*) 50 to 70 were used for the experiments. Mice were kept under standard housing conditions (12‐h/12‐h dark–light cycle, food and water ad libitum). Maintenance and handling of animals were performed according to EU and local governmental regulations. All experiments have been approved by the state of North Rhine Westphalia (Landesamt für Natur, Umwelt und Verbraucherschutz Nordrhein‐Westfalen, approval numbers 84‐02.04.2021.A189).

### 2.2 Preparation of Hippocampal and Cerebellar Slices

Transgenic NG2‐EYFPki mice (Karram et al. 2008) of postnatal days (*p*) 50 to 70 were anesthetized, sacrificed by decapitation and their brains were cut into 300 μm thick hippocampal coronal sections or into 250 μm thick cerebellar parasagittal sections with a vibratome (Leica, VT1200S). Slice preparation was performed at 6°C in a solution containing (in mM): 1.25 NaH_2_PO_4_, 87 NaCl, 2.5 KCl, 7 MgCl_2_, 0.5 CaCl_2_, 25 glucose, 25 NaHCO_3_, 61 sucrose (325–335 mOsm; preparation solution). Slices were then stored for 15 min in the same solution at 35°C and were subsequently transferred into artificial cerebrospinal fluid (aCSF) containing (in mM): 132 NaCl, 3 KCl, 2 MgCl_2_, 2 CaCl_2_, 10 glucose, 1.25 NaH_2_PO_4_, 20 NaHCO_3_ (305–315 mOsm; room temperature). Solutions were always gassed with carbogen.

### 2.3 Patch Clamp Recordings

Slices were then transferred to a recording chamber mounted on an upright microscope (Axioskop FS2, Zeiss, equipped with a CCD camera, VX45, Optronis, infrared‐DIC optics and epifluorescence, Polychrome II, Till Photonics), and constantly perfused with oxygenated aCSF. NG2 glial cells located in the hippocampal CA1 stratum radiatum or in the cerebellar molecular layer of the vermal lobule VI were identified by their intrinsic EYFP fluorescence and their characteristic whole‐cell current patterns during patch clamp recordings. Membrane currents were amplified (EPC 800, HEKA elektronik, Lambrecht, Germany), filtered at 1, 3 or 10 kHz and sampled at 10 kHz. The resistance of the patch pipettes ranged from 2 to 4 MΩ. Series resistance compensation was used to improve voltage clamp control. For all experiments, the pipette solution was composed of (in mM): 120 CsCl, 2 MgCl_2_, 0.5 CaCl_2_, 5 BAPTA, 10 HEPES, 3 Na_2_‐ATP, 10 TEA. Series and membrane resistance were monitored at constant intervals during the recordings. The membrane capacitance was determined from the mean integral (Δ*Q*) under the capacitive current elicited by 10 consecutive voltage steps from −70 to −60 mV. To analyze PSCs, stimulation of Schaffer collaterals (SCs) or CFs was performed through a chlorinated silver electrode inserted in a low‐resistance (< 1 MΩ), aCSF‐filled glass capillary serving as a monopolar stimulation electrode. Stimulation sequences were generated with an AM‐Systems isolation pulse stimulator (model 2100, Jerusalem, Israel). For paired‐pulse recordings, double pulses with an inter‐stimulus interval of 50 ms were applied every 15 s. In the hippocampus, the stimulation electrode was positioned in the CA1 stratum radiatum, close to the patch electrode, and moved under optical control at around 60 μm distance to optimize EPSC responses. In the cerebellum, the stimulation electrode was placed in the granule cell layer at a distance of 60–200 μm from the patch electrode. Recordings were obtained at 35°C. Picrotoxin was always present in the bath solution. Stimulation pulse duration was 100 μs while pulse intensity was adjusted to obtain postsynaptic responses alternating with failures. For characterization of AMPAR currents with intracellular Naspm (100 μM), K^+^ and Na^+^ channel blockers (100 μM quinine, 100 μM BaCl_2_, 0.5 μM TTX) and a GABA_A_ receptor antagonist (100 μM picrotoxin) were added to the recording solution. AMPAR currents were evoked by bath application of 250 μM kainate.

### 2.4 Fluorescence‐Activated Cell (FAC) Sorting and RT‐qPCR

Mice (NG2ki‐EYFP, p60, male and female) were sacrificed, their brains were dissected and whole hippocampi were isolated under microscopic control (Stereo microscope, Zeiss, Germany). Cell suspensions were prepared by mincing the tissue after digestion in papain and DNaseI (37°C, 25 min, Neural Dissociation Kit, Miltenyi, Germany). NG2 glia were identified by their EYFP fluorescence (emission at 527 nm) and sorted by an FACSAriaIII flow cytometer (70 μm nozzle, BD Biosciences, Heidelberg, Germany) into tubes containing Hanks' balanced salt solution (HBSS, without Ca^2+^ and Mg^2+^). After centrifugation (2000 g, 10 min) the supernatant was discarded and the cells were suspended in 200 μL lysis/binding buffer (Invitrogen, Darmstadt, Germany), frozen in liquid nitrogen and stored at −80°C. Messenger RNA was obtained from isolated cells after cell lysis by using oligo(dT)25‐linked Dynabeads (Invitrogen). The beads with adherent mRNA were suspended in DEPC‐treated water (20 μL). For first strand synthesis, reverse transcription (RT) was performed using oligo‐dT_24_‐primers (5 μM, Eurogentec, Seraing, Belgium). The reaction mix was incubated for 1 h at 50°C (final volume 40 μL; see also Hardt et al. 2021). The reaction mixture for real‐time PCR contained Takyon real‐time PCR mastermix (Eurogentec) and Taqman primer/probe mix (Thermo Fisher Scientific, Darmstadt, Germany). 1 μL of the RT product was added, the reaction volume was 12.5 μL. PCRs for the respective target genes and β‐actin, as a housekeeping gene, were run in parallel wells for each sample, respectively, and triplicates for each sample were performed. Water served as a negative control in each run. After denaturation (95°C, 10 min), 50 PCR cycles were performed (denaturation at 95°C, 15 s; primer annealing and extension at 60°C, 60 s; thermocycler CFX 384, Biorad, Munich, Germany). Fluorescence intensity was read out during each annealing/extension step. The target gene/β‐actin gene expression ratio was determined by comparing C_T_ values of the target gene with those of the reference gene. The relative quantification of different genes was determined according to the 2^ΔΔCT^ method:

(1) $$X_{\text{target}}/X_{\beta -\text{actin}}=E^{\mathrm{CT}\beta -\text{actin}}/E^{\text{CTtarget}}$$

yielding gene ratios with X being the input copy number and C_T_ the cycle number at threshold. The amplification efficiency for TARP γ2 was 1.89, for γ4: 1.90, for γ7: 1.92, for γ8: 1.96, for CNIH‐2: 1.98, and for β‐actin: 1.94. The expression ratios for the AMPAR subunits were determined according the 2^ΔΔCT^ method.

### 2.5 Data Analysis

EPSCs were analyzed with custom‐written macros in Igor Pro 8 software (Wavemetrics, USA). Stimulus artifacts were removed offline by subtracting averaged failure traces. The rectification index (RI) was determined by comparing the membrane conductance at −70 mV and + 40 mV according to equation:

(2) $$\mathrm{RI}=\left[I_{+40\mathrm{mV}}/\left(+40\mathrm{mV}-E_{\mathrm{rev}}\right)\right]/\left[I_{-70\mathrm{mV}}\left(-70\mathrm{mV}-E_{\mathrm{rev}}\right)\right]$$

Data were tested for normal distribution by the Shapiro–Wilk test, and with the Levene's test for homogeneity of variance. Subsequently, a paired‐sample or two‐sample Student's *t*‐test with or without Welch's correction, depending on the variances, was performed. Non‐parametric data were tested with the Mann–Whitney U‐test. Parametric data are given as mean ± SD and represented by bar plots. Median value and quartiles (25%–75%) are given in case of non‐parametric data, which are represented by box plots. “*N*” and “*n*” refer to the number of mice and cells, respectively. Significant differences were indicated by *(*p* < 0.05), **(*p* < 0.01), ***(*p* < 0.001), and ****(*p* < 0.0001).

## 3 Results

### 3.1 EPSCs of NG2 Glia in the Cerebellum Are More Strongly Influenced by CP‐AMPARs Than in the Hippocampus

**FIGURE 1:**
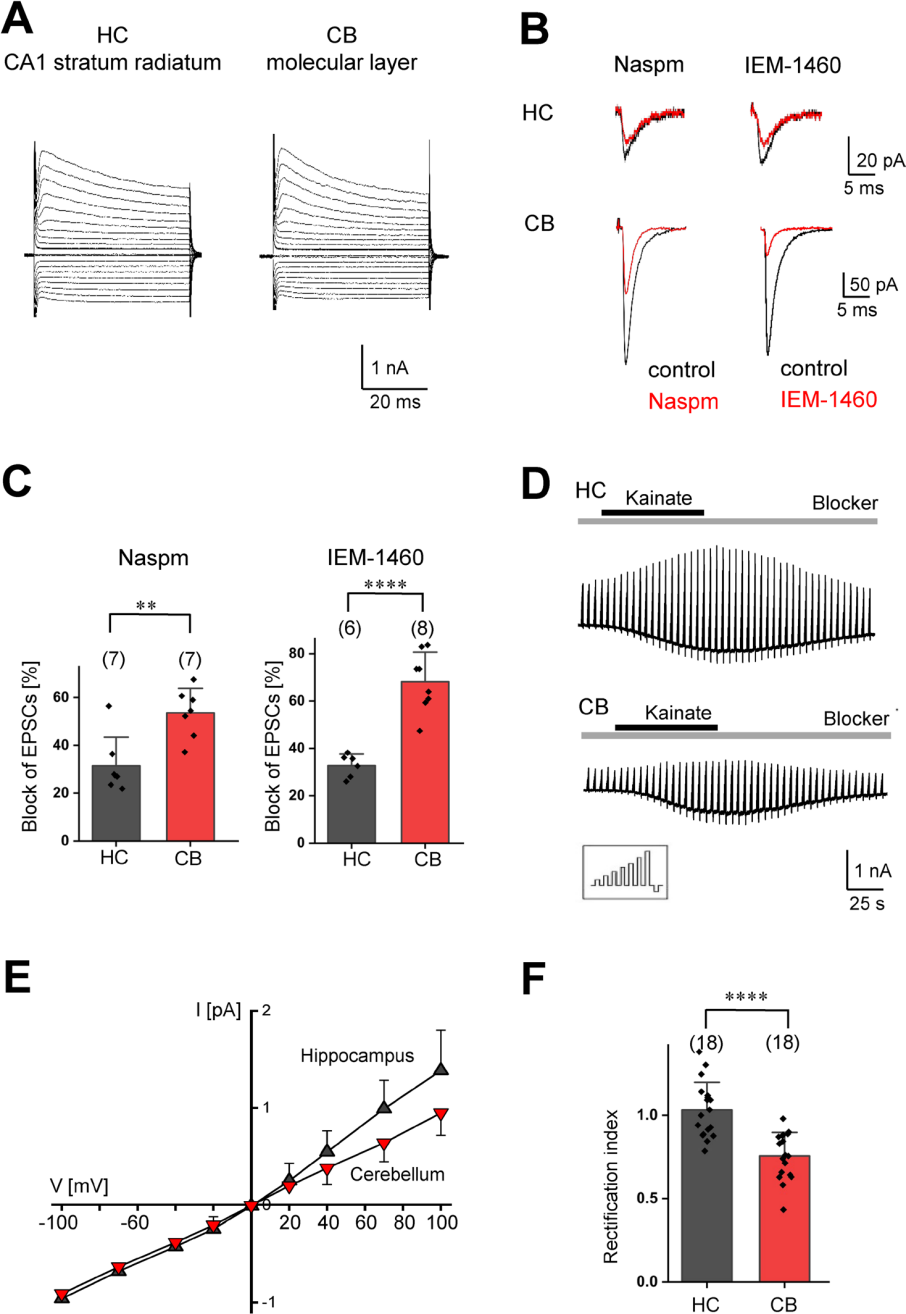
Block of CP‐AMPARs by polyamine derivatives in hippocampal and cerebellar NG2 glia. (A) Representative current patterns of NG2 glial cells in acute slices obtained from the CA1 stratum radiatum and from the molecular layer of the vermal lobule VI (p60). Cells were de‐ and hyperpolarized between −160 and +20 mV (10 mV increment, holding potential −80 mV; KCl‐based intracellular solution). (B) Evoked EPSCs elicited in NG2 glia upon stimulation of SCs in the CA1 region of the hippocampus (HC, upper panels, average of 40 EPSCs each) and CFs in the cerebellar molecular layer (CB, lower panels, average of 44 and 26 EPSCs, respectively). Responses were partially blocked by extracellularly applied polyamines (Naspm, 50 μM, left; IEM‐1460, 100 μM; right; red traces; membrane potential −70 mV). Bath solution contained picrotoxin (100 μM) and CTZ (100 μM). (C) Summary of EPSC inhibition by Naspm and IEM‐1460 in NG2 glia (Naspm, left panel: 31.5 ± 10.8 vs. 53.6% ± 10.2%, *n* = 7 *N* = 4, two‐sample *t*‐test, *p* = 0.003; IEM‐1460, right panel: 32.8% ± 4.8% vs. 68.1% ± 12.5%, *n* = 6 *N* = 5, two‐sample *t*‐test *p* = 0.00003). (D) Membrane currents were elicited in NG2 glia of the hippocampus (upper panel) and cerebellum (lower panel) by de‐ and hyperpolarizing voltage steps from −70 mV to −40, −20, 0, 20, 40, 70, 100, and −100 mV; duration 100 ms, interval 100 ms, every 4.5 s (cf. inset). The pipette solution contained Naspm (100 μM) while the extracellular solution was supplemented with quinine (200 μM), BaCl_2_ (100 μM), TTX (0.5 μM) and picrotoxin (100 μM) (gray bar). Kainate (250 μM) was added to the bath as indicated (black bar). (E) Average current–voltage relationships of kainate‐induced responses (cf. D) in hippocampal and cerebellar NG2 glia. Currents before and during agonist application were subtracted at corresponding voltages. Responses were normalized to maximum inward currents and averaged (hippocampus, black, *n* = 18; cerebellum, red, *n* = 18). (F) Rectification indices differed significantly between both regions (hippocampus: 1.03 ± 0.17, *n* = 18, *N* = 3; cerebellum: 0.76 ± 0.14, *n* = 18, *N* = 3; two sample *t*‐test *p* < 0.00006). CB: Cerebellum, HC: Hippocampus. Cell numbers are presented in parentheses. Significant differences are indicated by asterisks.

NG2 glia are present throughout the hippocampus and cerebellum, but they occur at a lower density in the latter region (Degen et al. 2012). To select NG2 glia in the slices for recording, the intrinsic fluorescence signal of NG2ki‐EYFP mice was used (Karram et al. 2008). Several previous studies have characterized the morphological and electrophysiological properties of these cells in the cerebellum and hippocampus (e.g., Degen et al. 2012; Moshrefi‐Ravasdjani et al. 2017; Seifert and Steinhäuser 2018). Typically, the whole cell current pattern of NG2 glial cells is characterized by depolarization‐induced small Na^+^ inward and prominent A‐type K^+^ outward currents while hyperpolarization activates large inwardly rectifying K^+^ currents (Moshrefi‐Ravasdjani et al. 2017; Seifert and Steinhäuser 2018). Qualitatively, the current patterns of these cells look similar in the hippocampus and cerebellum, and no brain region‐dependent differences were found regarding input resistance and resting membrane potential (Figure 1A; cf. below).

The contribution of CP‐AMPARs to evoked EPSCs was evaluated by extracellular application of two CP‐AMPAR blockers: 1‐naphthyl acetyl spermine (Naspm, Koike et al. 1997) and IEM‐1460 (Samoilova et al. 1999). Both blockers have a hydrophobic head that binds to the central cavity of the ion channel pore and a polyamine tail anchoring to the selectivity filter (Twomey et al. 2018). These blockers can only enter the pore when the channel is in the open state, remaining trapped inside until the channel opens again. To better visualize receptor currents, AMPAR desensitization was inhibited using cyclothiazide (CTZ, 100 μM), while GABA_A_ receptors were blocked with picrotoxin (100 μM). CTZ reliably increased the peak amplitude of evoked EPSCs and prolonged current decay (cf. also Figure 4C).

In the hippocampus, paired‐pulse stimulation (0.6 Hz, interpulse interval of 50 ms) of SCs triggered inward currents in NG2 glial cells located in the stratum radiatum of the CA1 region as previously reported (*V*_h_: −70 mV, Bergles et al. 2000; Jabs et al. 2005). The mean EPSC amplitude after the first pulse was −37.3 ± 8.7 pA (range −14 to −56 pA, *n* = 19, *N* = 11) (Figure 1B, upper panel). Synaptic currents showed rapid kinetics (20%–80% rise time: 0.56 ± 0.12 ms; monoexponential current decay, time constant 2.69 ± 0.91 ms) and a paired‐pulse ratio (PPR) of 2.03 ± 0.44 (not shown). Application of Naspm (50 μM) reduced the current amplitudes by about 32% (Figure 1B,C), which was in line with earlier observations (Hardt et al. 2021). In the cerebellum, we placed the electrode in the granule cell layer to stimulate CFs and evoke EPSCs in NG2 glia located in the molecular layer (Lin et al. 2005). Here, the average EPSC amplitude was −338.9 ± 291 pA (range −85 to −1106 pA, *n* = 19, *N* = 13; Figure 1B, lower panel). Rise times of EPSCs were shorter than in the hippocampus (0.40 ± 0.08 ms; two‐sample *t*‐test *p* < 0.0001) while current decayed more slowly (3.83 ± 1.80 ms; *p* < 0.05). At CF‐NG2 glia synapses we observed paired‐pulse depression (PPD; PPR = 0.56 ± 0.11; not shown). EPSC block by Naspm and IEM‐1460 was significantly stronger than in the hippocampus (Figure 1B,C). In cerebellar NG2 glia, the IEM‐1460 mediated block of synaptic currents was more efficient than inhibition by Naspm (*p* < 0.05; Figure 1C). Altogether, these results show that both in hippocampal and cerebellar NG2 glia, Naspm and IEM‐1460 partially inhibited synaptic currents through CP‐AMPARs. However, the impact of postsynaptic CP‐AMPAR in cerebellar NG2 glial cells is significantly higher compared to hippocampal NG2 glia.

The stronger effect of CP‐AMPAR blockers in the cerebellum suggested different AMPAR subunit compositions between both brain regions. Besides the Ca^2+^ impermeable GluA2 subunit, auxiliary subunits, such as TARPs, can also influence the binding of polyamines to AMPAR complexes (Cull‐Candy and Farrant 2021). However, recent studies indicated that intracellular application of Naspm effectively blocks CP‐AMPARs, regardless of TARP expression (Coombs et al. 2023). Thus, we added Naspm (100 μM) to the intracellular solution and isolated AMPAR currents by co‐applying Na^+^ and K^+^ channel blockers as well as a GABA_A_ receptor antagonist (quinine, 200 μM; Ba^2+^,100 μM; TTX, 0.5 μM; picrotoxin, 100 μM). In the hippocampus, the input resistance increased from 60 MΩ (50–96 MΩ) to 1048 MΩ (735–1169 MΩ), and the resting membrane potential depolarized from −92 mV (median; quartiles: −89 to −93 mV) to −60 mV (−53 to −69 mV). Under this condition, AMPARs in NG2 glial cells were activated through bath application of kainate (250 μM), eliciting inward currents of −14 ± 4 pA/pF (*V* = −70 mV, *n* = 18, *N* = 3; Figure 1D). In the cerebellum, the channel blockers depolarized NG2 glia from −86 mV (−75 to −93 mV) to −60 mV (−44 to −56 mV) and increased the input resistance from 75 MΩ (53 to 106 MΩ) to 1104 MΩ (882 to 1395 MΩ). Kainate induced inward currents of −12 ± 5 pA/pF (*V* = −70, *n* = 18, *N* = 3). With 100 μM Naspm in the recording pipette, we observed a slightly outwardly rectifying current–voltage relationship of AMPAR currents in hippocampal NG2 glia, while in the cerebellum inward rectification was observed (Figure 1E,F). Again, these data confirmed the higher extent of CP‐AMPAR expression in NG2 glia of the cerebellum compared to the hippocampus.

### 3.2 Hippocampal NG2 Glia Show Higher Expression of GluA2, TARPs γ4, 7, 8 and CNIH‐2

**FIGURE 2:**
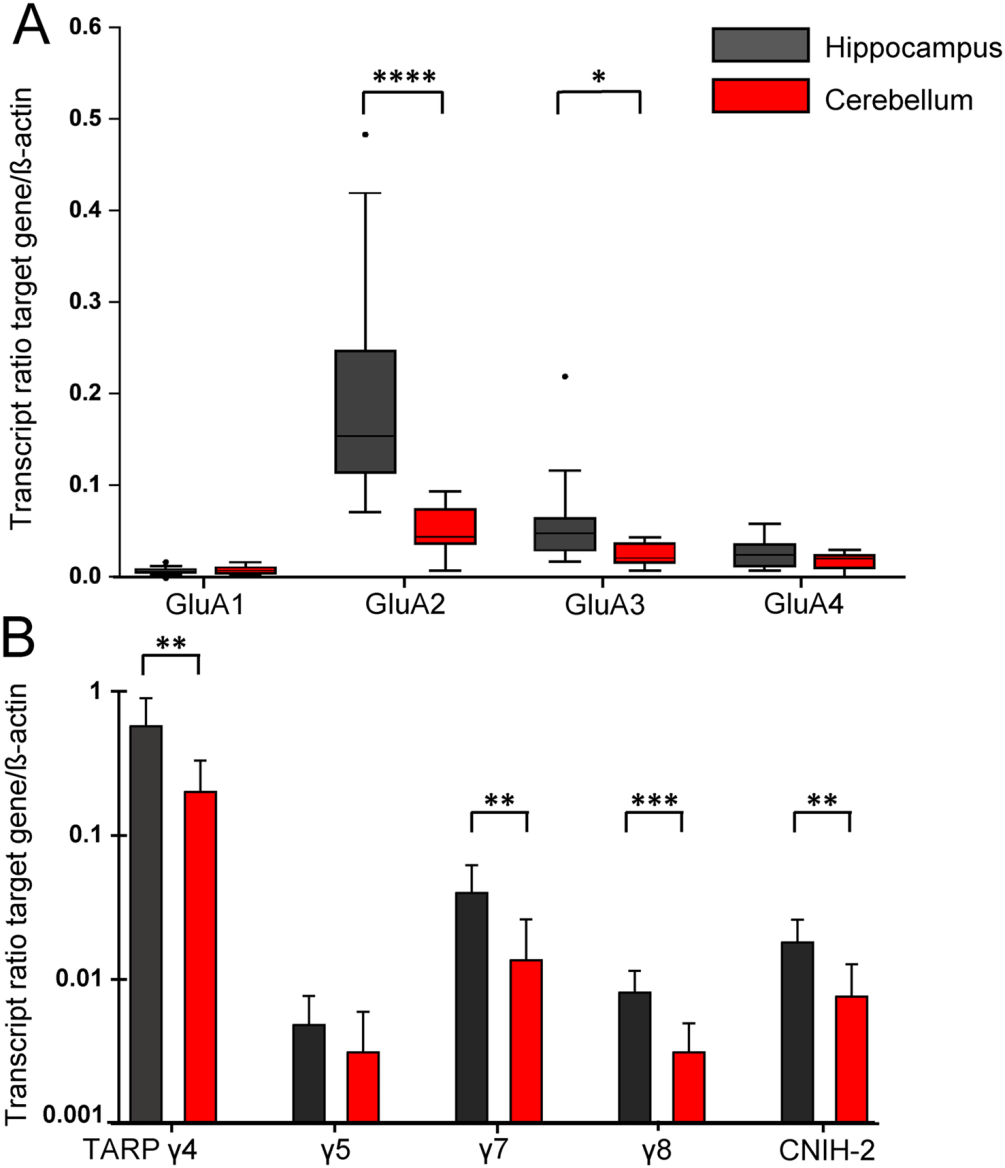
Transcript expression profiles of AMPARs in hippocampal (black) and cerebellar (red) NG2 glia. (A) Expression ratios (target genes to β‐Actin) of GluA1‐4 in FAC‐sorted NG2 glia, determined according to Equation (1). GluA1: HC, 0.007 (median; quartiles 0.006–0.010, *N* = 11); CB, 0.008 (0.005–0.012, *N* = 10). GluA2: HC, 0.155 (0.115–0.248, *N* = 12); CB, 0.045 (0.037–0.075, *N* = 11, *p* = 0.00006). GluA3: HC, 0.049 (0.030–0.065, *N* = 11); CB, 0.022 (0.017–0.037, *N* = 10, *p* = 0.016). GluA4: HC, 0.025 (0.013–0.037, *N* = 12); CB, 0.021 (0.011–0.025, *N* = 11). Mann–Whitney U‐test, box plots showing median (central line), quartiles (25 and 75%; box) and whiskers (1.5 times the interquartile range). (B) Expression ratios of auxiliary subunits. TARP γ4: HC, 0.570 ± 0.327, *N* = 12; CB, 0.201 ± 0.123, *N* = 8; *p* = 0.002. TARP γ5: HC, 0.005 ± 0.003, *N* = 12; CB, 0.004 ± 0.003, *N* = 6. TARP γ7: HC, 0.040 ± 0.022, *N* = 12; CB, 0.014 ± 0.013, *N* = 8; *p* = 0.007. TARP γ8: HC, 0.008 ± 0.003, *N* = 12; CB, 0.003 ± 0.002, *N* = 8; *p* = 0.0006. CNIH‐2: HC, 0.018 ± 0.008, *N* = 12; CB, 0.008 ± 0.005, *N* = 8; *p* = 0.006. Two‐tailed *t*‐test, bar graphs represented mean ± SD, number of animals in parentheses. Significant differences are indicated by asterisks. CB: Cerebellum, HC: Hippocampus. Data representing TARP and CNIH expression in the hippocampus (red bars in B) were taken from Hardt et al. (2021).

To evaluate if cerebellar and hippocampal NG2 glia differ in their AMPAR subunit composition, we harvested and FAC sorted NG2 glia from the hippocampus and cerebellum, isolated mRNA and compared receptor expression by real‐time RT‐qPCR using β‐actin as a housekeeping gene. The GluA2 subunit was much less abundant in cerebellar vs. hippocampal NG2 glia. GluA1 and GluA4 were similarly expressed in the two brain regions, while GluA3 transcript levels were higher in the hippocampus (Figure 2A).

AMPARs are accompanied by auxiliary subunits, which modulate receptor kinetics, polyamine‐binding and association with CP‐AMPAR subunits, for example, TARP γ2 (Jackson and Nicoll 2011; Zonouzi et al. 2011). We compared the expression pattern of auxiliary subunits in the cerebellum with that in the hippocampus, which we have reported previously (cf. Hardt et al. 2021). In both regions, TARP γ4 was most abundant, followed by TARP γ7 and CNIH‐2. They were all expressed more strongly in hippocampal NG2 glia. As expected, γ8 transcript levels were also higher in the hippocampus (Figure 2B). In contrast, TARP γ2 was less frequently detected in the hippocampus (in 3/12 samples) compared to the cerebellum (6/8 samples; Chi^2^‐test *p* < 0.05; not shown).

### 3.3 Larger Synaptic Glutamate Transients Contribute to a Higher Efficacy of CF‐NG2 Glia Synapses

**FIGURE 3:**
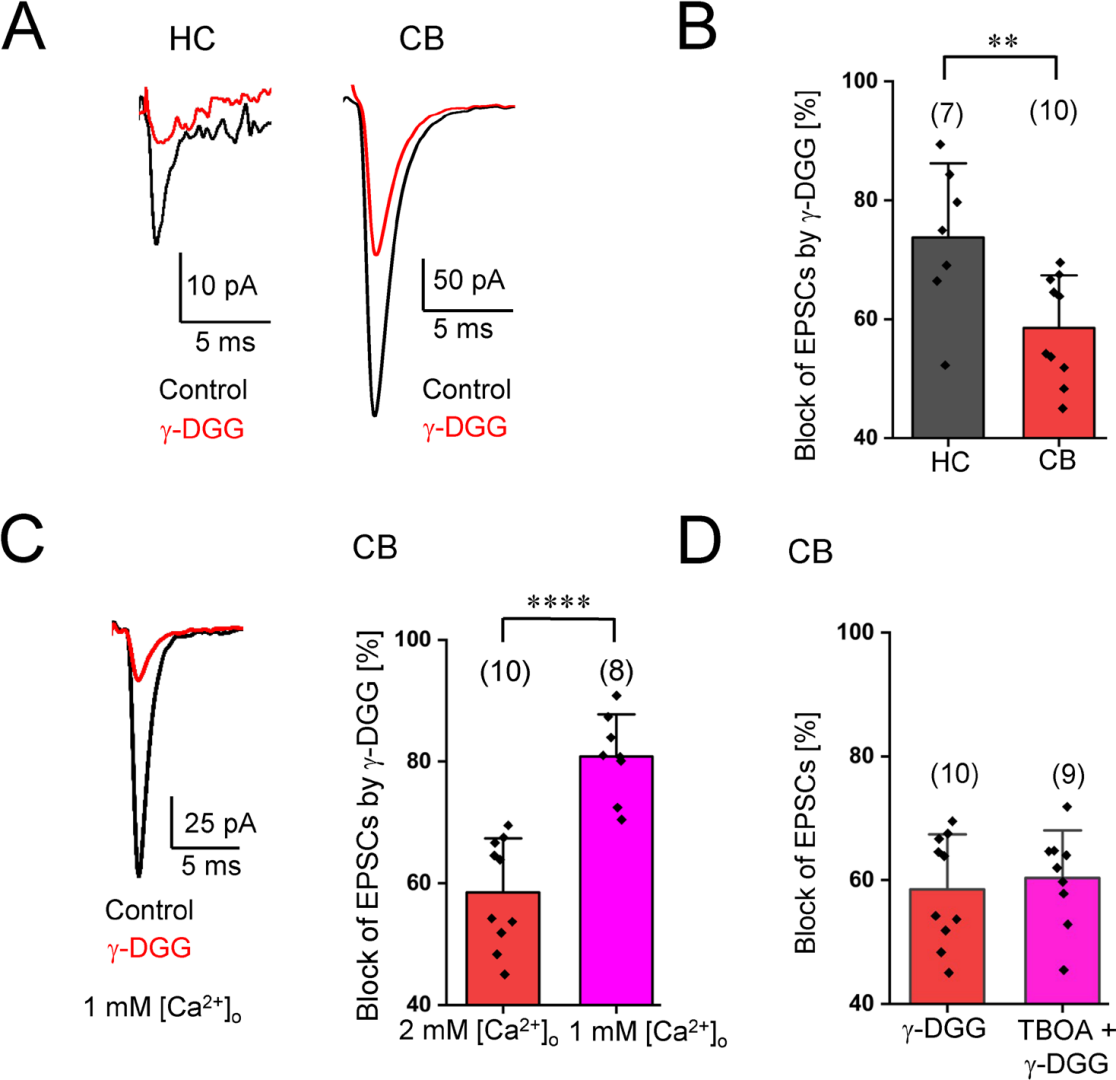
Multivesicular release influences the efficacy of CF‐NG2 glia synapses. (A) Representative EPSCs (membrane potential = −70 mV) obtained under control conditions (black traces) and after adding γ‐DGG (1 mM, red) in hippocampal (left, average of 16 EPSCs) and cerebellar (right, average of 40 EPSCs) NG2 glia. (B) The γ‐DGG induced reduction of EPSCs in the hippocampus (by 73.7% ± 12.4%; *n* = 7, *N* = 4) is much larger than in the cerebellum (58.5% ± 8.8%; *n* = 10, *N* = 5; two sample *t*‐test *p* = 0.009). (C) Left: Representative EPSCs during control condition (black trace, average of 32 EPSCs) and after application of γ‐DGG (red, average of 16 EPSCs) (1 mM [Ca^2+^]_o_; cerebellar slices). Right: Reducing [Ca^2+^]_o_ from 2 mM (favoring high P_r_) to 1 mM (low P_r_) enhanced the blocking effect of γ‐DGG on EPSCs in cerebellar NG2 glia (80.8% ± 6.8%; *n* = 8, *N* = 4) (two sample *t*‐test *p* = 0.00002). Data represent the ratios between mean peak current amplitudes after and before application of γ‐DGG. (D) DL‐TBOA (30 μM) did not further decrease EPSCs in cerebellar NG2 glia recorded in γ‐DGG (γ‐DGG + DL‐TBOA: Block by 60.3% ± 7.7%; *n* = 9, *N* = 6). Cell numbers are given in parentheses. Significant differences are indicated by asterisks.

The larger proportion of GluA2‐lacking AMPARs in the cerebellum may produce a higher efficacy of NG2 glia synapses because of the larger single channel conductance of CP‐AMPARs (Swanson et al. 1997). However, other factors additionally might influence glutamatergic signaling, for example, larger glutamate transients in CF‐NG2 glia synaptic clefts. To test this hypothesis, we applied the low‐affinity AMPAR antagonist, γ‐D‐glutamylglycine (γ‐DGG, 1 mM), while stimulating NG2 glia in the hippocampus and cerebellum. Indeed, γ‐DGG blocked a larger EPSC fraction in the hippocampus compared to cerebellar NG2 glia synapses (Figure 3A,B). These results suggested that larger glutamate transients contribute to the higher efficacy of neuron–NG2 glia signaling in the cerebellum. Previous studies have shown that synapses with a high release probability (*P*_r_), like the ones between CFs and Purkinje cells, can release multiple vesicles at a time (Wadiche and Jahr 2001). To test whether this is true also for cerebellar NG2 glia synapses, we lowered the extracellular Ca^2+^ concentration ([Ca^2+^]_o_) from 2 to 1 mM, while [Mg^2+^]_o_ was increased from 2 to 3 mM, and stimulated CFs. Under these conditions, which should lower the presynaptic *P*_r_, γ‐DGG blocked the EPSCs by about 80%, that is, much more efficiently than in the presence of 2 mM [Ca^2+^]_o_ (Figure 3C). Thus, glutamate transients at low *P*_r_ were significantly smaller than under control conditions. It is therefore likely that under control conditions, a high *P*_r_ triggered multivesicular release, which increased glutamate concentration transients in the synaptic cleft.

CF‐Purkinje cell synapses are almost entirely covered and isolated from each other by Bergmann glia (Xu‐Friedman and Regehr 2004) while no Bergmann glia have been detected at CF‐NG2 glia synapses (Lin et al. 2005). To test whether glutamate uptake affects synaptic currents, we used the glutamate transport blocker, DL‐TBOA (30 μM). After stimulating CF‐NG2 glia synapses for 10 min, we applied γ‐DGG (1 mM) w/wo the transport blocker. DL‐TBOA slightly increased the decay time constant of the EPSCs by 16% ± 10% (control: 1.02 ± 0.23 ms; in the presence of TBOA: 1.16 ± 0.26 ms; paired *t*‐test *p* < 0.05, *n* = 10, *N* = 6; not shown) but EPSC amplitudes were similarly blocked by γ‐DGG w/wo DL‐TBOA (Figure 3D). Thus, it is likely that the lower sensitivity to γ‐DGG of cerebellar NG2 glia synapses resulted from larger glutamate transients due to multivesicular transmitter release.

### 3.4 Short‐Term Plasticity at CF‐NG2 Glia Synapses Depends on Pre‐ and Postsynaptic Mechanisms

**FIGURE 4:**
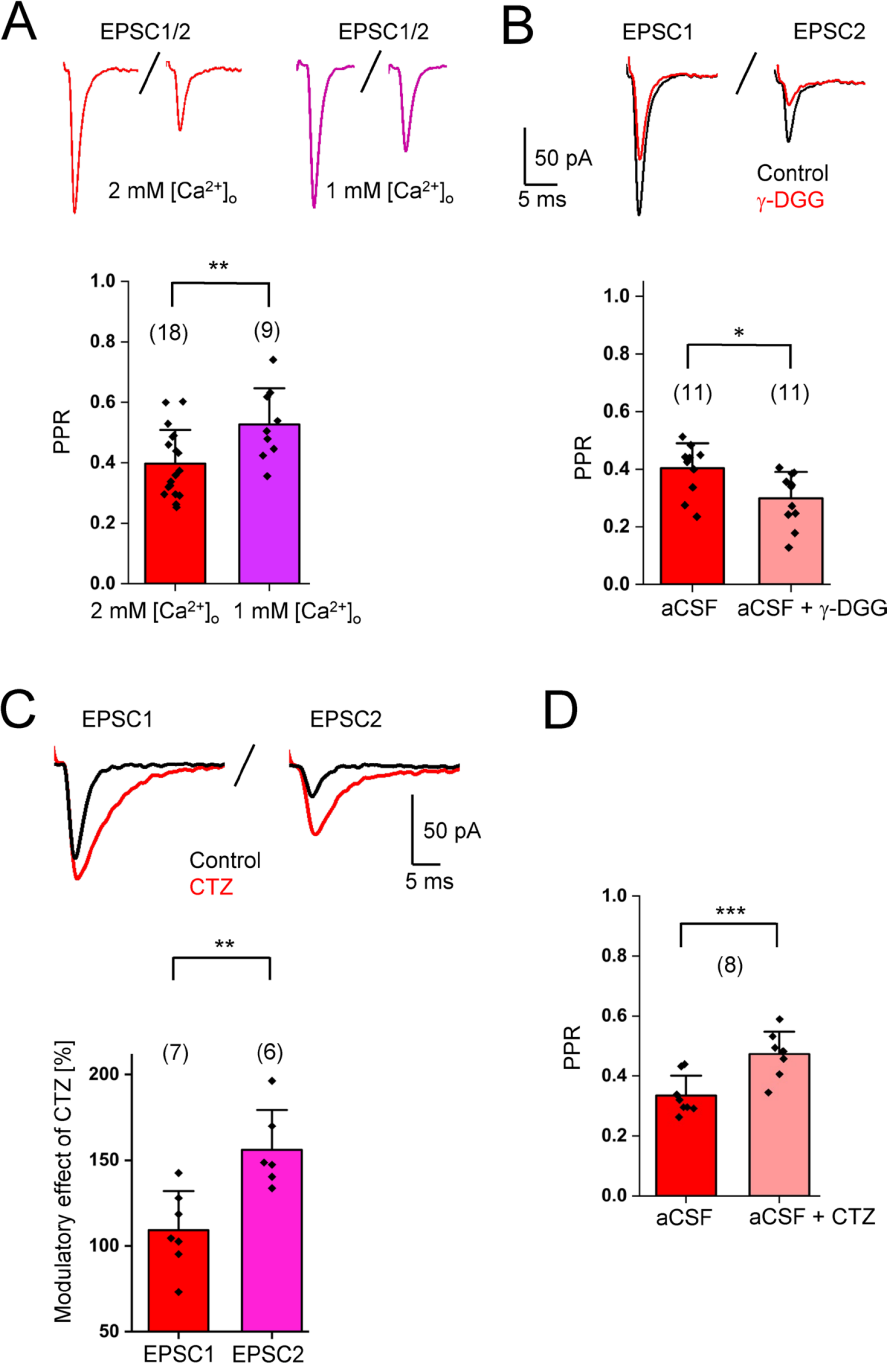
Short‐term plasticity at cerebellar CF‐NG2 synapses. (A) Top: Representative EPSCs evoked by paired pulses in 2 mM [Ca^2+^]_o_ (red, average of 33 EPSCs) and 1 mM [Ca^2+^]_o_ (purple, average of 16 EPSCs) (interstimulus interval 50 ms). Bottom: The PPR was Ca^2+^ dependent (2 mM [Ca^2+^]_o_: 0.40 ± 0.11; *n* = 18, *N* = 6; 1 mM [Ca^2+^]_o_: 0.53 ± 0.11; *n* = 9, *N* = 4; two‐sample *t*‐test *p* = 0.01). (B) Top: EPSCs from paired pulse stimulation (interstimulus interval 50 ms) under control conditions (black) and after application of γ‐DGG (red). Traces show averages of 35 EPSCs. Bottom: γ‐DGG (1 mM) reduced the PPR (control: 0.40 ± 0.08; *n* = 11, *N* = 6; in γ‐DGG: 0.30 ± 0.09; *n* = 11, *N* = 6, *p* = 0.03). (C) Top: Paired‐pulse EPSCs before (black) and after application of CTZ (red) (interstimulus interval 50 ms). Traces show averages of 23 EPSCs Bottom: Modulatory effect of CTZ (50 μM) on EPSCs evoked in NG2 glia after stimulation of CFs. Data represent the ratio of the mean peak amplitudes after and before CTZ application (EPSC1: 109.2% ± 22.8%; *n* = 7, *N* = 3; EPSC2: 156.0% ± 23.2%; *n* = 6, *N* = 3; *p* = 0.003). (D) CTZ increased the PPR at CF‐NG2 glia synapses (control: 0.33 ± 0.06; CTZ: 0.47 ± 0.07; *n* = 8, *N* = 4; paired‐sample *t*‐test *p* = 0.0009). Cell numbers are given in parentheses. Significant differences are indicated by asterisks.

Short‐term plasticity at CF‐Purkinje cell synapses is mostly mediated by presynaptic mechanisms (Hashimoto and Kano 1998; Zucker and Regehr 2002). At CF‐NG2 glia synapses, the PPR significantly increased in low [Ca^2+^]_o_, even though PPD was still clearly evident (Figure 4A). This suggested that the PPR of the cerebellar CF‐NG2 glia synapse was dependent on presynaptic *P*_r_ (Regehr 2012). Moreover, γ‐DGG decreased the PPR (Figure 4B), which also hinted at presynaptic mechanisms underlying short–term plasticity at this synapse, probably due to larger glutamate transients and less efficient γ‐DGG block upon the first pulse (Rabl et al. 2006). One of these mechanisms might be vesicle depletion, which has been traditionally used to explain PPD at many synapses, including the CF‐Purkinje cell synapse (Liley and North 1953; Betz 1970; Zucker and Regehr 2002). We also checked whether postsynaptic receptor desensitization contributed to PPD at glial synapses. Therefore, we stimulated CF‐NG2 glia synapses in the presence of 50 μM CTZ to prevent rapid receptor desensitization. As shown in Figure 4C, the modulatory effect of CTZ was significantly higher for EPSC2 than for EPSC1. Accordingly, PPD strongly decreased (Figure 4D). These results, taken together, indicate that at CF‐NG2 glia synapses both pre‐ and postsynaptic mechanisms contribute to short‐term plasticity. Notably, these results differ from those obtained at CF‐Purkinje neuron synapses where desensitization did not have a role in short‐term plasticity (Dittman and Regehr 1998).

## 4 Discussion

In this study, we compared the properties of AMPARs in NG2 glia from the hippocampus and cerebellum, identifying key molecular and functional differences. We observed a higher Ca^2+^ permeability of AMPARs in cerebellar NG2 glia, which is likely due to a lower GluA2 expression together with an increased presence of TARP γ2. Our data also suggest that multivesicular glutamate release from CFs contributes to the higher synaptic efficacy of cerebellar NG2 glia. Finally, we examined short‐term plasticity at CF‐NG2 glia synapses and found that both pre‐ and postsynaptic mechanisms are involved.

### 4.1 Regional Differences of Ca^2+^ Permeability of AMPARs

CFs in the cerebellum innervate NG2 glia, making hundreds of synaptic contacts (Lin et al. 2005). In the hippocampus, glutamatergic innervation of NG2 glia is much lower, and EPSCs are 10‐fold smaller (Bergles et al. 2000; Jabs et al. 2005; Haberlandt et al. 2011). This stronger innervation is just one of the factors explaining the higher responsiveness of cerebellar NG2 glia. Indeed, differences in the effects of the Naspm or IEM‐1460 shown here indicated a distinct expression of CP‐AMPARs in NG2 glia of both brain regions. Extracellular polyamines selectively bind to the pore of CP‐AMPAR subunits, composed of GluA1, GluA3, and GluA4, while the presence of GluA2 limits the Ca^2+^ permeability of the receptor and prevents polyamine binding (Washburn et al. 1997; Bowie and Mayer 1995). Nevertheless, a prevalence of CP‐AMPARs might be masked by the action of TARP‐γ2, which can reduce spermine potency by reshaping the selectivity filter (Brown et al. 2017; Coombs et al. 2023). Intracellular application of polyamine derivatives can circumvent the blockage caused by TARPs and allow polyamine binding. In our study, replacing spermine with Naspm in the intracellular solution led to a complete block of outward currents through CP‐AMPARs, independently of auxiliary subunits. These functional analyses proved that in the hippocampus and cerebellum, CP‐AMPARs differently contributed to the EPSCs. Our molecular analyses were in line with these conclusions. Expression of GluA2, which determines the Ca^2+^ permeability of the receptors (Geiger et al. 1995; Swanson et al. 1997), was much lower in cerebellar NG2 glia while TARPγ2 transcript levels were higher. The latter is not only essential for membrane trafficking of CP‐AMPARs (Zonouzi et al. 2011) but it also influences receptor gating and pharmacological properties, enhancing synaptic strength and stability (Tomita et al. 2005; Milstein and Nicoll 2009). Additionally, TARPs γ4, 7, 8 and CNIH‐2 were less abundant in cerebellar NG2 glia compared to the hippocampal counterpart, which might contribute to differences in pharmacology and kinetics of AMPARs in both regions (Greger et al. 2017).

The AMPAR composition is developmentally regulated and varies across cell types and brain regions (Schwenk et al. 2014; Lalanne et al. 2016; Matta et al. 2013). For example, cerebellar cells display a distinct subunit expression profile influencing synaptic transmission and plasticity (Bats et al. 2013). Such diversity in AMPAR subunit composition, which is regulated by TARPs, is also seen in other CNS regions where the presence or absence of specific subunits regulates receptor function and synaptic plasticity (Kumar et al. 2002; Clem and Huganir 2010). In hippocampal NG2 glia, the Ca^2+^ permeability of AMPARs increases with age (Hardt et al. 2021) but at the same developmental stage, this permeability is much larger in the receptors of cerebellar NG2 glia. This difference might account for larger EPSC amplitudes in the latter because the single channel conductance of CP‐AMPARs exceeds that of AMPAR complexes containing the Ca^2+^ impermeable subunit GluA2 (Swanson et al. 1997). Notably, the rapid kinetics and high conductance of CP‐AMPARs may enable NG2 glia to effectively detect and respond to the strong synaptic input from CFs. Our experiments with γ‐DGG suggest a relatively high P_r_ at CF‐NG2 glia synapses, similar to what has been observed in CF‐Purkinje cell synapses (Wadiche and Jahr 2001). At CF‐NG2 glia synapses, reducing P_r_ increased the γ‐DGG‐mediated block of EPSCs, indicating multivesicular release which in turn produced larger glutamate transients than those generated in hippocampal SC‐NG2 glia synapses.

Thus, our experiments found clear differences in the properties of glutamatergic signaling to NG2 glia between the hippocampus and cerebellum, suggesting that the cells undergo region‐specific developmental regulation. Fate‐mapping studies revealed that NG2 glia in different CNS regions originate from distinct progenitor pools and are shaped by environmental cues (Viganò and Dimou 2016). The stronger Ca^2+^ permeability of AMPARs in cerebellar NG2 glia may not only enhance synaptic strength but also influence intracellular signaling pathways related to NG2 glia proliferation and differentiation. Moreover, recent evidence suggested that Ca^2+^ signaling in NG2 glia regulates brain‐derived neurotrophic factor (BDNF) levels, influencing neuronal plasticity and circuit function (Timmermann et al. 2023). Ca^2+^ permeable receptors in cerebellar NG2 glia may therefore not only enhance synaptic efficacy but also contribute to activity‐dependent release of neurotrophic factors, potentially affecting neuronal survival and synaptic remodeling. Understanding these mechanisms could provide insights into how NG2 glia shape neural circuits and respond to environmental stimuli.

### 4.2 Short Term Plasticity at CF‐NG2 Glia Synapses Depends on Pre‐ and Postsynaptic Mechanisms

Our results suggest that a high *P*_r_ at CF‐NG2 glia synapses caused significant PPD, resembling short‐term plasticity at CF‐Purkinje cell synapses (Dittman and Regehr 1998; Hashimoto and Kano 1998; Zucker and Regehr 2002). We observed decreased PPD after reduction of [Ca^2+^]_o_, hinting at a presynaptic, Ca^2+^‐dependent mechanism, and the application of CTZ also increased PPR. This molecule is an allosteric modulator of AMPARs, which eliminates rapid desensitization, enhances EPSC amplitudes and slows down the decay of AMPAR–mediated PSCs (Yamada and Tang 1993). At the concentration used, CTZ did not interfere with presynaptic transmitter release (Xu‐Friedman and Regehr 2003; Yang and Xu‐Friedman 2008). Therefore, the effect of CTZ on PPD suggested a postsynaptic mechanism regulating short‐term plasticity at CF‐NG2 glia synapses. Notably, CTZ had no effect on PPD at Purkinje cell synapses (Dittman and Regehr 1998; Hashimoto and Kano 1998). Possibly, structural distinctions between CF–Purkinje cell and CF‐NG2 glia synapses or the absence of Bergmann glia coverage contribute to these differences (Xu‐Friedman and Regehr 2004). Thus, although NG2 glia and Purkinje cells share the same presynaptic fibers (Lin et al. 2005), the presynaptic activity is differently transmitted and transformed at the neuronal and glial synapses.

## Author Contributions

Experiments were performed in the Institute of Cellular Neurosciences I, Medical Faculty, University of Bonn, Bonn, Germany. **D.T.:** acquisition, analysis and interpretation of data, drafting the work; **N.G.:** acquisition and analysis of data; **R.J.:** analysis and interpretation of data; **C.H.:** interpretation of data, revising the manuscript; **C.S.:** conception and design, analysis and interpretation of data, drafting and revising the manuscript; **G.S.:** conception and design, acquisition, analysis and interpretation of data, drafting and revising the manuscript. All authors approved the final version of the manuscript and agree to be accountable for all aspects of the work in ensuring that questions related to the accuracy or integrity of any part of the work are appropriately investigated and resolved. All authors qualify for authorship, and all those who qualify for authorship are listed.

## Funding

This work was supported by Deutsche Forschungsgemeinschaft (grants STE 552/5, SE 774/6).

## Ethics Statement

Mice were kept under standard housing conditions (12/12 h light/dark cycle, food, and water ad libitum). Maintenance and handling of animals were performed according to EU and local governmental regulations. All experiments have been approved by the state of North Rhine Westphalia (LANUV, veterinary license 84‐02.04.2021.A189).

## Conflicts of Interest

The authors declare no conflicts of interest.

## Data Availability

The data that support the findings of this study are available from the corresponding author upon reasonable request.
